# A 13-hour laboratory school study of lisdexamfetamine dimesylate in school-aged children with attention-deficit/hyperactivity disorder

**DOI:** 10.1186/1753-2000-3-17

**Published:** 2009-06-09

**Authors:** Sharon B Wigal, Scott H Kollins, Ann C Childress, Liza Squires

**Affiliations:** 1 University of California, Irvine, Child Development Center, Irvine, California, USA; 2 Duke University Medical Center, Durham, North Carolina, USA; 3 Center for Psychiatry and Behavioral Medicine, Las Vegas, Nevada, USA; 4 Shire Development Inc, Wayne, Pennsylvania, USA

## Abstract

**Background:**

Lisdexamfetamine dimesylate (LDX) is indicated for the treatment of attention-deficit/hyperactivity disorder (ADHD) in children 6 to 12 years of age and in adults. In a previous laboratory school study, LDX demonstrated efficacy 2 hours postdose with duration of efficacy through 12 hours. The current study further characterizes the time course of effect of LDX.

**Methods:**

Children aged 6 to 12 years with ADHD were enrolled in a laboratory school study. The multicenter study consisted of open-label, dose-optimization of LDX (30, 50, 70 mg/d, 4 weeks) followed by a randomized, placebo-controlled, 2-way crossover phase (1 week each). Efficacy measures included the SKAMP (deportment [primary] and attention [secondary]) and PERMP (attempted/correct) scales (secondary) measured at predose and at 1.5, 2.5, 5, 7.5, 10, 12, and 13 hours postdose. Safety measures included treatment-emergent adverse events (AEs), physical examination, vital signs, and ECGs.

**Results:**

A total of 117 subjects were randomized and 111 completed the study. Compared with placebo, LDX demonstrated significantly greater efficacy at each postdose time point (1.5 hours to 13.0 hours), as measured by SKAMP deportment and attention scales and PERMP (*P *< .005). The most common treatment-emergent AEs during dose optimization were decreased appetite (47%), insomnia (27%), headache (17%), irritability (16%), upper abdominal pain (16%), and affect lability (10%), which were less frequent in the crossover phase (6%, 4%, 5%, 1%, 2%, and 0% respectively).

**Conclusion:**

In school-aged children (6 to 12 years) with ADHD, efficacy of LDX was maintained from the first time point (1.5 hours) up to the last time point assessed (13.0 hours). LDX was generally well tolerated, resulting in typical stimulant AEs.

**Trial registration:**

Official Title: A Phase IIIb, Randomized, Double-Blind, Multi-Center, Placebo-Controlled, Dose-Optimization, Cross-Over, Analog Classroom Study to Assess the Time of Onset of Vyvanse (Lisdexamfetamine Dimesylate) in Pediatric Subjects Aged 6–12 With Attention-Deficit/Hyperactivity Disorder.

ClinicalTrials.gov Identifier: NCT00500149

## Background

Stimulants are the mainstay of pharmacotherapy for attention-deficit/hyperactivity disorder (ADHD). Their safety and efficacy have been well documented [1-3]. Within this class of medications, amphetamine and methylphenidate are the most widely prescribed agents for the treatment of ADHD [4]. An early double-blind, parallel-group study of dexamphetamine in 38 children with hyperkinetic disorder demonstrated efficacy for dexamphetamine with 62% considered greatly improved overall vs 17% for placebo [5]. Target symptoms such as hyperactivity and distractibility were also significantly improved by treatment. The adverse events associated with dexamphetamine in this study were those typically seen with stimulants, including reduction in appetite, weight loss, insomnia, stomach ache and changes in emotional expression [5]. A second arm of the study with methylphenidate found substantial similarity between the 2 medications in efficacy and safety. In the years since, immediate- and sustained-release formulations of d-amphetamine have been used extensively to treat ADHD with a number of reports concluding that there is a high degree of similarity in efficacy and tolerability between d-amphetamine and methylphenidate and that differences are subtle and often subject-specific ([1,6-8]. There is an important clinical need for long-acting stimulant medications with efficacy beyond 12 hours' duration among children with ADHD who require symptom control that extends into the later hours of the day [9].

Lisdexamfetamine dimesylate (LDX; Vyvanse^®^; Shire US Inc) is a prodrug stimulant indicated for the treatment of ADHD. LDX is a therapeutically inactive molecule that subsequently upon ingestion is hydrolyzed by endogenous enzymes to l-lysine, a naturally occurring essential amino acid, and active d-amphetamine, which is responsible for its therapeutic effect [10]. Preliminary nonclinical data suggest that conversion of LDX to d-amphetamine and l-lysine may also occur in the blood [11]. The conversion of LDX to d-amphetamine is unlikely to be affected by gastrointestinal pH and variations in normal gastrointestinal transit times [12].

Clinical studies of LDX have been completed in school-aged children (6–12 years) with ADHD. A 6-week, randomized, double-blind, crossover study in a laboratory school setting [13] similar to the current study showed that LDX was significantly more effective in reducing ADHD symptoms compared with placebo (*P *< .0001), as measured by the Swanson, Kotkin, Agler, M-Flynn, and Pelham deportment (SKAMP-D) subscale throughout the day [14]. The SKAMP-D (see methods section for further details on SKAMP and all subscales) was used to assess behavioral manifestations of ADHD during analog classroom sessions [14]. Efficacy in LDX-treated subjects compared with placebo was significant beginning at 2 hours postdose and lasted up to 12 hours postdose, the last time point measured. LDX was generally well tolerated with adverse events (AEs) similar to those seen with other once-daily stimulants [13].

A second 4-week, multicenter, randomized, double-blind, placebo-controlled, parallel-group trial in children with ADHD evaluated the efficacy and safety of LDX (30, 50, and 70 mg/d) over 4 weeks of treatment [10]. Significant improvements in ADHD Rating Scale Version IV (ADHD-RS-IV) [15] scores were noted for all doses of LDX compared with placebo (all, *P *< .001) [10]. LDX produced significant improvement in ADHD symptom control as early as the first week of treatment, compared with placebo (*P *< .001) [10]. Also parent ratings of their child's response to treatment, measured by the ADHD Index on the Conners' Parent Rating Scale (CPRS) [16], were improved and maintained at each time point throughout the day (*P *< .001 vs placebo and vs baseline at approximately 10 AM, 2 PM, and 6 PM). As in the previous study, LDX was generally well tolerated, with an AE profile similar to that of other extended-release stimulant products [10].

The present study replicated and expanded upon the findings of the previous laboratory school study described above, sharing methodologic similarities such as age of subjects and use of a laboratory school protocol. Thus, the onset of efficacy was investigated as early as 1.5 hours and duration of efficacy was measured up to 13 hours following dosing.

### Study objectives

The primary objective of this study was to assess the initial onset of efficacy of LDX compared with placebo, as measured by the SKAMP-D subscale (questions 5 through 8 on the SKAMP scale), in children aged 6 to 12 years with ADHD. The key secondary objective was to assess the duration of efficacy of LDX compared with placebo, also using the SKAMP-D subscale. Additional secondary assessments of efficacy over time included the Permanent Product Measure of Performance (PERMP), SKAMP attention (SKAMP-A) subscale (questions 1 through 4), the SKAMP quality of work subscale (questions 9 through 11), the SKAMP total score, the ADHD-RS-IV, and Clinical Global Impressions (CGI) scale. The study also evaluated the safety of LDX through assessment of AEs, vital signs, electrocardiograms (ECGs), and physical examination.

## Materials and methods

This randomized, double-blind, multicenter, placebo-controlled, dose-optimization, crossover, laboratory school study of LDX was conducted at 7 study sites in the United States. Subjects were recruited from June through December 2007. All study activities were performed in accordance with the principles of the International Conference on Harmonization Good Clinical Practice, 18th World Medical Assembly (Helsinki 1964), and amendments of the 29th (Tokyo 1975), the 35th (Venice 1983), the 41st (Hong Kong 1989), and the 48th (South Africa 1996) World Medical Assemblies.

### Study participants

This study enrolled boys and girls aged 6 to 12 years who satisfied *Diagnostic and Statistical Manual of Mental Disorders, Fourth Edition, Text Revision *(DSM-IV-TR) [17] criteria for a primary diagnosis of ADHD, combined or hyperactive-impulsive subtype. Subjects were also required to have a baseline ADHD-RS-IV score ≥ 28, age-appropriate intellectual functioning as determined by an intelligence quotient (IQ) of ≥ 80 on the Kaufman Brief Intelligence Test [18], the ability to complete the PERMP assessment, and blood pressure within the 95th percentile for age, gender, and height.

Key exclusion criteria were presence of a comorbid psychiatric condition with severe symptoms, conduct disorder, or other medical condition that could confound assessments, pose a risk to the subject, or prohibit study completion. Other exclusion criteria were adverse reaction or nonresponsiveness to previous amphetamine therapy, pregnancy, substance abuse, weight < 22.7 kg (50 lb), body mass index > 98th percentile for age, seizure within the last 2 years, tic or Tourette disorder, use of medication with central nervous system effects (excluding bronchodilators), or clinically significant laboratory and ECG abnormalities. Children whose current ADHD medication provided effective control of symptoms with acceptable tolerability were also excluded.

### Study design

**Figure 1:**
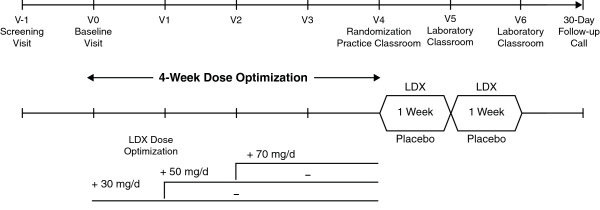
**Study design**. V: visit; LDX: lisdexamfetamine dimesylate.

The study consisted of a screening phase (approximately 3 weeks), washout if applicable (up to 1 week, depending on the subject's current medication), open-label, stepwise dose optimization (4 weeks), double-blind, crossover treatment with weekly assessments in a laboratory school setting (2 weeks), and safety follow-up by telephone (30 days) (Figure 1). The use of some medications was prohibited during the study due to their potential to interfere with safety, efficacy, or tolerability assessments (norepinephrine reuptake inhibitors; investigational compounds; antipsychotic, anxiolytic, or sedative-hypnotic medications; antidepressants; clonidine; antihypertensive agents; psychostimulants; and sedating antihistamines). Except for stimulant medications and sedating antihistamines (which were discontinued up to 7 days before baseline), use of these medications up to 30 days prior to screening was also prohibited.

Subjects were required to visit the clinic at screening (visit -1), baseline (visit 0), dose optimization (visits 1 through 4, corresponding to weeks 1 through 4), and double-blind treatment (visits 5 and 6, which were analog classroom sessions in the laboratory school setting). Visit 6 also served as the end-of-study visit.

### Administration of study drug

Following screening and washout, eligible subjects entered the open-label dose-optimization phase, during which they began receiving LDX followed by evaluation for efficacy and tolerability of that dosage approximately 7 days later. Dosage was initiated at 30 mg/d LDX and adjusted to the next available dose at weekly intervals, until optimal dose was reached. Optimal dose was defined as the dose that produced a reduction in ADHD-RS-IV score ≥ 30% and CGI-Improvement (CGI-I) score of 1 or 2 and had tolerable side effects. Tolerability was determined by the investigator, based on review of AEs and clinical judgment. Once reached, the optimal dose was maintained for the remainder of the dose-optimization phase and was used for the double-blind treatment sequence period. Clinicians could increase the current dose to provide additional symptom control. One dose reduction was permitted if subjects experienced unacceptable tolerability of the current dose. Subjects were discontinued if they were unable to tolerate LDX or had not reached their optimal dose by visit 4. The dose dispensed at visit 3 was the dose used during the double-blind treatment sequence period. During visit 4, subjects attended a half-day practice laboratory school with analog classroom sessions to become familiar with classroom schedules and procedures. SKAMP assessments were performed, and 3 to 5 practice PERMP tests were given during the practice session.

Following dose optimization, subjects entered the 2-week double-blind treatment period. Subjects were randomized to receive daily LDX treatment (at the optimized dose) for 1 week followed by daily placebo capsules (identical in appearance to LDX capsules) for 1 week, or vice versa. For the first 6 days of each week during double-blind treatment, study drug was administered by the parent. On the last day of each week, with subject having taken LDX or placebo for the preceding 6 days according to their randomization schedule, the daily dose was administered by study staff in the laboratory school at the start of the analog classroom assessment day.

Ideally, each session had a cohort of 10 to 16 participants; however, classroom size could be increased to 18 subjects with prior approval from the sponsor. Cohort size ranged from 6 subjects to 17 subjects with most between 11 and 15 subjects. Two of the 7 study sites enrolled 1 cohort each with fewer than 10 subjects (6 and 8, respectively); 3 of the 7 sites enrolled 1 cohort each of 13 to 17 subjects; and 2 of the 7 sites enrolled 2 cohorts each of 11 to 17 subjects each. At visits 5 and 6 (laboratory school days), the subjects arrived at 6 AM, and assessments of SKAMP and PERMP were taken at 0.5 hours predose (6:30 AM). Subjects then received their randomized treatment (7 AM). SKAMP and PERMP assessments were performed during analog classroom sessions as noted below. SKAMP assessments were performed by observers who were provided with training to help ensure reliability. The laboratory school day ended at approximately 8:30 PM.

A follow-up telephone call was made approximately 30 days after the subject's last dose of study drug to collect information on any ongoing or new AEs, serious AEs, and concomitant medications. Appropriate follow-up was continued until the investigator judged that all safety concerns were resolved.

### Outcome measures

#### Efficacy

##### Primary efficacy measure

The primary efficacy measure was the SKAMP-D subscale. The SKAMP scale is a validated rating scale that assesses manifestations of ADHD in a classroom setting through several subscales, including deportment (behavior) and attention [14]. SKAMP assessments were conducted during a half-day practice of the laboratory school visit (visit 4). During the full-day visits (visits 5 and 6), SKAMP assessment times were 0.5 hours predose and at 1.5, 2.5, 5, 7.5, 10, 12, and 13 hours postdose. Multiple SKAMP assessments were completed at the end of individual classroom sessions across the day by observers who rated each subject on 13 items, using a 7-point impairment scale (0 = normal, 6 = maximal impairment). In this study, SKAMP-D comprised 4 of the 13 items on the SKAMP scale: interacting with other children, interacting with adults, remaining quiet according to classroom rules, and staying seated according to classroom rules [14,19]. SKAMP-D scores were calculated as the mean of the ratings for these 4 items at each time point of each visit. Mean SKAMP-D scores at visits 5 and 6 were also calculated as the mean of the ratings for these 4 items over all postdose time points of each visit.

##### Secondary efficacy measures

The PERMP, a 5-page math test consisting of 80 problems per page (total of 400 problems) [19], was used in this study to evaluate effortful performance in the classroom as a measure of efficacy. Subjects were instructed to work at their seats and to complete as many problems as possible in 10 minutes. The appropriate level of difficulty for each student was determined previously based on results of a math pretest administered at screening. Performance was evaluated using two scores: PERMP-A (number of problems attempted) and PERMP-C (number of problems correct). The PERMP was completed during analog classroom sessions at the same time points as the SKAMP scale. To avoid taking the same test more than once during the study, subjects received randomized problems in a different version of the test at each assessment.

The SKAMP-A subscale is a measure of attention and comprises the following 4 items on the SKAMP scale: getting started on assignments, sticking with tasks, attending to an activity, and making activity transitions [14,19]. The SKAMP quality of work subscale comprises 3 items: completing assigned work, performing work accurately, and being careful and neat while writing or drawing. The scores for SKAMP-A, SKAMP quality of work, and SKAMP total were calculated as the mean of the ratings for the items making up the score at each time point of each visit.

The ADHD-RS-IV [15] is a clinician-rated scale that reflects current symptoms of ADHD based on DSM-IV-TR criteria; it is a global assessment that measures the severity of symptoms from visit to visit, but is not being utilized to assess symptoms of ADHD over the course of the day. The ADHD-RS-IV consists of 18 items that are grouped into 2 subscales (hyperactivity/impulsivity and inattention). Each item is scored on a 4-point scale from 0 (no symptoms) to 3 (severe symptoms), yielding a total score of 0 to 54. The ADHD-RS-IV was administered at baseline and each visit thereafter to assess efficacy.

The CGI [20] provides a global evaluation of baseline severity and improvement over time, and, similarly as the ADHD-RS-IV scale does, measures global impressions of severity from visit to visit but not over the course of the day. At baseline, the investigator used the CGI-Severity (CGI-S) to rate severity on a scale that ranged from 1 (normal, not at all ill) to 7 (among the most extremely ill subjects) plus a not assessed option. At each visit thereafter, the clinician used the CGI-I to rate improvement relative to baseline on a scale ranging from 1 (very much improved) to 7 (very much worse) plus a not assessed option. For analysis, CGI-I scores were dichotomized so that very much improved (CGI-I score of 1) and much improved (CGI-I score of 2) were combined into 1 category (improved), and the remaining responses were combined into the other category (not improved). CGI-I scores of 0 (not assessed) were not included in the analysis.

#### Safety

AEs, concomitant medications, and vital signs (including systolic blood pressure [SBP], diastolic blood pressure [DBP], and pulse) were recorded at each visit. ECGs were conducted at screening (visit -1), baseline (visit 0), and the end-of-study visit (visit 6). A physical examination was conducted at screening and the end-of-study visit. Clinical laboratory tests (including hematology, chemistry, and urinalysis) were conducted only at screening. Treatment-emergent AEs (TEAEs), referring to events with onset after the first date of treatment, and no later than 3 days following termination of treatment, were recorded separately for the dose-optimization phase and the double-blind laboratory school phase of the study.

### Statistical analyses

Determination of sample size for the primary comparison of time of onset of LDX versus placebo was based on analysis of SKAMP-D scores from a previous crossover study [13] as well as other analog classroom design studies in ADHD. Assuming a standard deviation (SD) of 0.95 (the maximum SD reported in the previous LDX crossover study) and based on an average difference in SKAMP-D scores between placebo and LDX of 0.50 units for hours 1 and 2 in the previous study, 96 subjects (48 subjects in each treatment sequence) would need to complete the study to detect a true difference of 0.50 units in mean SKAMP-D scores between placebo and LDX at 95% power when testing at a significance level of α = .05 (2-sided). However, 128 subjects were planned for enrollment, since as many as 25% of subjects were predicted to discontinue based on the proportion of subjects discontinuing prematurely in prior LDX studies. All statistical tests were 2-sided and performed at the 0.05 significance level.

#### Efficacy

The primary population for efficacy assessments was the intent-to-treat population (ITT) population, which consisted of all randomized subjects who received at least 1 dose of study medication with at least 1 postrandomization measurement of the primary efficacy variable (mean SKAMP-D score over the course of a day) available for analysis.

The primary efficacy measure was SKAMP-D subscale score at each time point and mean score throughout the day. The primary objective was to assess time of onset of LDX compared with placebo as measured by SKAMP-D, with a key secondary objective to assess duration of efficacy of LDX using this subscale. A linear mixed model was used to analyze the mean SKAMP-D score as well as the SKAMP-D scores for each time point. In this model, the fixed effects were sequence, period, and treatment, while the random effect was subject-within-sequence. Raw means, least-squares (LS) means, differences in LS means, and 95% confidence interval (CI) for the difference between treatment groups, *P *values, and model results were calculated for each postdose time point and for mean score over the treatment day for the ITT population.

A post hoc analysis examined change from predose for SKAMP-D, PERMP-A, PERMP-C, and SKAMP-A. Other post hoc analyses also examined SKAMP-D scores by optimized dose and by study site. Postdose SKAMP-D scores were analyzed by t-test on the change from predose within group. Potential site and treatment interactions were examined using the primary model with investigative-site and site-by-treatment interactions added as factors at the significance level of 0.10.

Since the study had a crossover design and assessed duration of efficacy, the last observation carried forward (LOCF) method was not a valid approach for data that were incomplete because of discontinuation or unavailability. Therefore, incomplete data due to these reasons were set as missing for purposes of statistical analysis.

#### Safety

Safety data for the dose-titration period were analyzed using combined data from all subjects in the safety population (defined as those subjects who entered the dose-titration period and received open-label treatment). Safety data from the double-blind sequence period were analyzed using data from each treatment group where applicable in the randomized population (defined as all randomized subjects who received at least 1 dose of study medication during the double-blind crossover period). Safety summaries for vital signs were presented by visit for each treatment group in the safety population. Safety summaries for ECGs were presented for the baseline and end-of-study visits, for all subjects in the safety population combined. For each AE, frequency was calculated by treatment group and for number and percentage of subjects who reported the event. Continuous variables related to these safety assessments were summarized using the number of observations, mean, SD, minimum, median, and maximum values, while categorical (nominal) variables were summarized using number of observations and percentages.

## Results

### Subjects

A total of 129 subjects were enrolled and entered the open-label, dose-optimization phase (Table 1). Of these, 117 (90.7%) were randomized to the double-blind crossover phase, 113 (87.6%) were included in the ITT population, and 111 (86.0%) completed the study. Four of the 117 subjects in the randomized population were not included in the ITT population because they did not have at least 1 SKAMP-D score available after randomization. Mean (SD) age of the safety population was 10.1 (1.5) years, and mean (SD) weight was 72.8 (17.3) lb. The safety population was made up of 76.0% (n = 98) male subjects and 70.5% (n = 91) Caucasians. At baseline, all subjects were diagnosed with the combined ADHD subtype and had a mean (SD) ADHD-RS-IV total score of 42.4 (7.1). According to the prespecified statistical analysis plan, efficacy analyses were based on the ITT population of 113 subjects and safety analyses were based on the safety population of 129 subjects.

**Table 1:** Subject Demographics (Safety Population) and Disposition

**Subject Category**			**LDX Optimal Dose**		
	
		**30 mg/d**	**50 mg/d**	**70 mg/d**	**All Doses**
Safety population		58 (100.0)	50 (100.0)	21 (100.0)	129 (100.0)

Age (years)	Mean (SD)	9.8 (1.5)	10.2 (1.3)	10.4 (1.9)	10.1 (1.5)

Gender					
Male	n (%)	44 (75.9)	37 (74.0)	17 (81.0)	98 (76.0)
Female		14 (24.1)	13 (26.0)	4 (19.0)	31 (24.0)

Race					
Caucasian		38 (65.5)	37 (74.0)	16 (76.2)	91 (70.5)
Black or African American		11 (19.0)	4 (8.0)	2 (9.5)	17 (13.2)
Native Hawaiian or Other Pacific Islander	n (%)	0	1 (2.0)	0	1 (0.8)
Asian		0	0	0	0
American Indian or Alaska Native		2 (3.4)	0	0	2 (1.6)
Other		7 (12.1)	8 (16.0)	3 (14.3)	18 (14.0)

Ethnicity					
Hispanic or Latino	n (%)	9 (15.5)	11 (22.0)	6 (28.6)	26 (20.2)
Not Hispanic or Latino		49 (84.5)	39 (78.0)	15 (71.4)	103 (79.8)

ADHD-RS-IV Total Score at Baseline	Mean (SD)	40.5 (6.7)	43.4 (7.5)	45.7 (5.7)	42.4 (7.1)

Randomized population		46 (79.3)	50 (100.0)	21 (100.0)	117 (90.7)

Intent-to-treat population		46 (79.3)	47 (94.0)	20 (95.2)	113 (87.6)

Completed study		44 (75.9)	47 (94.0)	20 (95.2)	111 (86.0)

Reason for discontinuation*					
Adverse event^†^		8 (13.8)	1 (2.0)	0	9 (7.0)
Protocol violation		1 (1.7)	1 (2.0)	0	2 (1.6)
Consent withdrawn		3 (5.2)	1 (2.0)	1 (4.8)	5 (3.9)
Lost to follow-up		2 (3.4)	0	0	2 (1.6)
Lack of efficacy		0	0	0	0
Other		0	0	0	0

LDX: lisdexamfetamine dimesylate; ADHD-RS-IV: Attention-Deficit/Hyperactivity Disorder Rating Scale IV

*Includes all subjects who discontinued during dose-optimization and crossover periods based on optimized dose.

^†^All AEs leading to discontinuation occurred during dose optimization. Eight discontinuations occurred before randomization. These subjects were taking 30 mg/d LDX when the discontinuation-related AE occurred. One discontinuation occurred after randomization. This subject was taking 50 mg/d LDX when he experienced acute gastritis, causing him to miss the visit 4 practice day.

Of the 7 study sites, 1 site was also a site from the previous LDX analog classroom study. Per the principal investigator of that site, there was no subject overlap and, therefore, no subjects with prior exposure to LDX from clinical trials were included in this study [Previous exposure clarification. Personal Communication with AC. Childress on December 8, 2008].

Twelve of the 18 study discontinuations (Table 1) occurred during dose optimization while each subject was receiving 30 mg/d LDX (8 due to AEs, 1 due to protocol violation, and 3 due to withdrawal of consent). Six discontinuations occurred during the crossover phase: 2 while the subject was receiving LDX (protocol violation, withdrawal of consent), and 4 while the subjects were receiving placebo (loss to follow-up in 2 subjects, AE in 1 subject, and withdrawal of consent in 1 subject). No subject discontinued due to lack of efficacy of active treatment.

### Efficacy assessments

#### Primary measure

##### SKAMP-D

**Figure 2:**
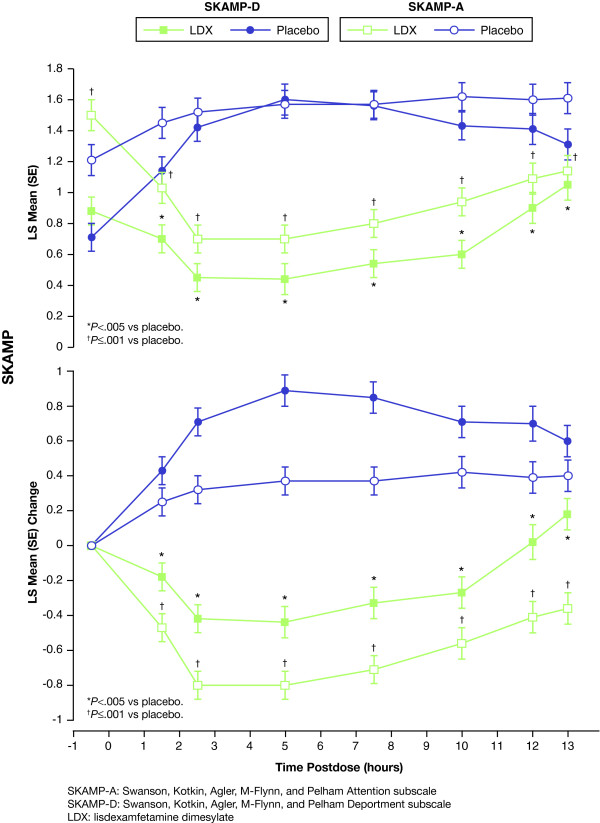
**Time course of SKAMP-D (closed symbols) and SKAMP-A (open symbols) assessment over the laboratory school day**. LS mean (SE) actual scores (top) and change from predose (bottom) for LDX (squares) and placebo (circles) at predose (-0.5 h) and at 1.5, 2.5, 5, 7.5, 10, 12, and 13 h postdose. Scores were compared using a linear mixed model with sequence, period, and treatment as fixed effects and subject within sequence as a random effect. Lower scores denote improvement. *Denotes *P *< .005 LDX compared with placebo for SKAMP-D. ^†^Denotes *P *≤ .001 LDX compared with placebo for SKAMP-A.

LDX demonstrated significant improvement on the SKAMP-D compared with placebo, at 1.5 hours (the first postdose time point measured; primary endpoint) and continuing through all time points up to and including 13.0 hours postdose (the last time point measured; key secondary endpoint). There was significant separation of LDX from placebo at all postdose time points for the SKAMP-D score analysis (as measured by LS mean [SE]; *P *< .005 for all time points) (Table 2) and [(Figure 2) top]. Analysis of change from predose [(Figure 2) bottom] was performed using the same statistical model that was used for analysis of the primary efficacy variable, SKAMP-D score, and was designed, together with the summary statistics for actual scores by time point to provide additional context. Post hoc analysis of SKAMP-D scores in the LDX group showed that significant improvement relative to predose was seen at all postdose time points except 12 and 13 hours. At 12 and 13 hours postdose, SKAMP-D scores in this group were numerically worse but not statistically different from predose levels. Conversely, SKAMP-D scores were significantly worse than predose at all postdose time points in the placebo group. Negative scores indicated improvement when reporting SKAMP changes. The differences in LS means (95% CI) of LDX vs placebo at 1.5 hours and 13.0 hours were -0.45 (-0.62, -0.28; *P *< .0001) and -0.26 (-0.43, -0.08; *P *< .005), respectively. The mean score difference in LS means (95% CI) of LDX vs placebo was -0.74 (-0.85, -0.63; *P *< .0001).

**Table 2:** LS Mean (SE) SKAMP Scores at Predose and 1.5 and 13.0 Hours Postdose*

	**SKAMP-D** **LS Mean (SE)**		**SKAMP-A** **LS Mean (SE)**		**SKAMP-Quality of Work** **LS Mean (SE)**		**SKAMP-Total** **LS Mean (SE)**	
	**LDX**	**Placebo**	**LDX**	**Placebo**	**LDX**	**Placebo**	**LDX**	**Placebo**

Predose	0.88 (0.09)	0.71 (0.09)	1.50 (0.10)^†^	1.21 (0.10)	2.90 (0.08)^†^	1.72 (0.08)	1.68 (0.07)^†^	1.22 (0.07)

1.5 hours	0.70 (0.09)^†^	1.14 (0.09)	1.03 (0.10)^†^	1.45 (0.10)	1.75 (0.09)	1.95 (0.09)	1.15 (0.08)^†^	1.62 (0.08)

13 hours	1.05 (0.10)^†^	1.31 (0.10)	1.14 (0.10)^†^	1.61 (0.10)	2.13 (0.10)^†^	2.46 (0.10)	1.43 (0.08)^†^	1.85 (0.08)

LS: least squares; LDX: lisdexamfetamine dimesylate; SKAMP: Swanson, Kotkin, Agler, M-Flynn, and Pelham scale; D: deportment; A: attention

*Lower SKAMP total and subscale scores are indicative of improvement.

^†^*P *< .005 vs placebo.

#### Secondary measures

##### PERMP-A and PERMP-C

**Figure 3:**
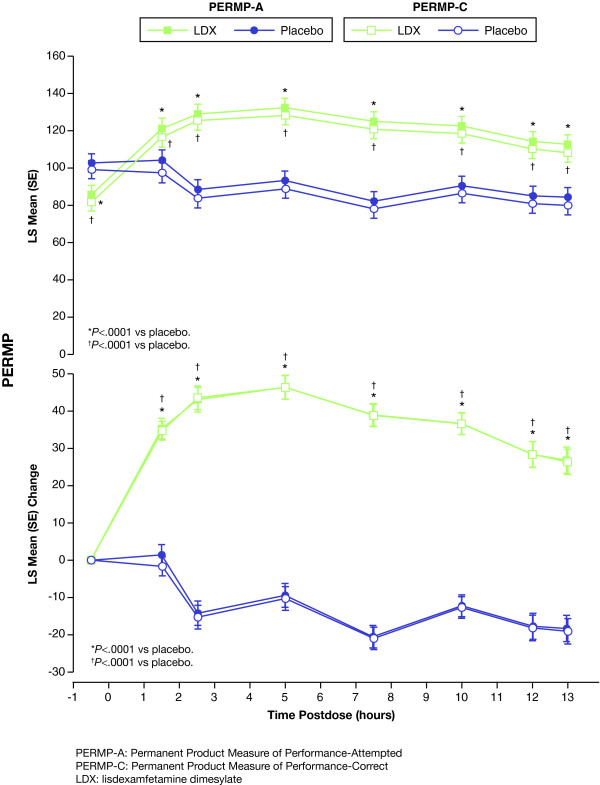
**Time course of PERMP-A (closed symbols) and PERMP-C (open symbols) assessment over the laboratory school day**. LS mean (SE) actual scores (top) and change from predose (bottom) for LDX (squares) and placebo (circles) at predose (-0.5 h) and at 1.5, 2.5, 5, 7.5, 10, 12, and 13 h postdose. Scores were compared using a linear mixed model with sequence, period, and treatment as fixed effects and subject within sequence as a random effect. Higher scores denote improvement. *Denotes *P *< .0001 LDX compared with placebo for PERMP-A. ^†^Denotes *P *< .0001 LDX compared with placebo for PERMP-C.

Results for PERMP-A and PERMP-C were also consistent with results from the SKAMP-D. For PERMP-A and PERMP-C, efficacy was shown at each postdose time point, at 1.5 hours and continuing up to and including 13.0 hours (Figures 3). LDX showed separation from placebo at all postdose time points for both the PERMP-A [(Figure 3), actual and change from predose, top and bottom, respectively] and PERMP-C [(Figure 3), actual and change from predose, top and bottom, respectively] score analyses (as measured by LS mean [SE]; *P *< .0001 for all time points). LS mean (SE) PERMP-A and PERMP-C scores for LDX groups [85.54 (4.88) and 81.86 (4.84), respectively] were significantly different from placebo groups [102.43 (4.88) and 99.17 (4.84), respectively; *P *< .005] at predose assessments (Figures 3). The differences in LS means (95% CI) of LDX vs placebo in PERMP-A at 1.5 hours and 13.0 hours were 16.97 (9.39, 24.56) and 28.28 (21.51, 35.04), respectively (both, *P *< .0001). The differences in LS means (95% CI) of LDX vs placebo in PERMP-C at 1.5 hours and 13 hours were 19.10 (12.25, 25.94) and 28.14 (21.46, 34.83), respectively (both, *P *< .0001).

##### SKAMP-A, SKAMP Quality of Work, and SKAMP Total Scores

Results from SKAMP-A were consistent with results from the primary efficacy measure (SKAMP-D). For SKAMP-A, LDX demonstrated significant efficacy compared with placebo at 1.5 hours (the first postdose time point measured) and continuing through all time points up to and including 13.0 hours postdose (the last time point measured). LDX showed complete separation from placebo at all postdose time points for the SKAMP-A score analysis (as measured by LS mean (SE) and LS mean change (SE) from predose; *P *≤ .001 vs placebo for all time points; Table 2 and [(Figure 2), top and bottom], respectively). Predose SKAMP-A scores were significantly different between the LDX group and placebo group (Table 2 and Figure 2). The differences in LS means (95% CI) of LDX vs placebo in SKAMP-A at 1.5 hours and 13 hours were -0.43 (-0.62, -0.23) and -0.47 (-0.62, -0.31), respectively (both, *P *< .0001).

Results of both SKAMP quality of work and SKAMP total scores (Table 2) were consistent with those seen with SKAMP-D. SKAMP quality of work subscale LS mean (SE) scores showed significant efficacy of LDX starting at the 2.5-hour time point (1.53 [0.09] LDX vs 2.42 [0.09] placebo;*P *< .0001). Significant efficacy compared with placebo continued at each postdose time point thereafter, up to and including 13.0 hours (all, *P *< .005). SKAMP total scores also showed significant efficacy compared with placebo at all points beginning at 1.5 hours and up to and including 13.0 hours (*P *< .0001). Predose SKAMP quality of work and total scores in the LDX groups were significantly different from those in the placebo groups (Table 2). The demonstration of efficacy at each postdose time point for SKAMP total scores was consistent with that seen for SKAMP-D and SKAMP-A.

### Dose analysis

Overall mean difference (95% CI) for placebo vs LDX was analyzed by optimized dose groups for SKAMP-D, SKAMP-A, SKAMP quality of work, and SKAMP total scores (Table 3), and these scores across optimized dose groups during the open-label, nonrandomized phase of this study were found to be consistent.

**Table 3:** Mean Difference in LS Means (95% CI) for SKAMP Scales by Optimized Dose Group*

**LDX Dose Group**	**SKAMP-D**	**SKAMP-A**	**Quality of Work**	**Total**
30 mg/d (n = 46)	-0.70	-0.59	-0.60	-0.73
	(-0.88, -0.52)	(-0.79, -0.40)	(-0.78, -0.42)	(-0.87, -0.59)

50 mg/d (n = 47)	-0.68	-0.61	-0.67	-0.74
	(-0.84, -0.52)	(-0.78, -0.45)	(-0.81, -0.54)	(-0.86, -0.62)

70 mg/d (n = 20)	-0.96	-0.89	-0.68	-0.99
	(-1.30, -0.63)	(-1.15, -0.64)	(-0.94, -0.42)	(-1.24, -0.74)

LDX: lisdexamfetamine dimesylate; LS: least squares; SKAMP: Swanson, Kotkin, Agler, M-Flynn, and Pelham scale; D: deportment; A: attention; CI: confidence interval

*Negative scores indicate improvement relative to placebo.

### Site analysis

The differences in LS mean (95% CI) for SKAMP-D scores for placebo vs LDX treatment by investigative site were all in the direction of improvement with LDX compared to placebo. Differences in LS mean (95% CI) scores between placebo and LDX ranged from -0.47 (-0.70 to -0.24) to -1.04 (-1.35 to -0.72). Variability between investigative sites in mean predose SKAMP-D scores was apparent but this did not seem to unduly influence postdose scores. Statistical interaction model analysis with investigative site and site by treatment added as factors found no significant interaction at the 0.10 level (*P *= .153).

#### ADHD-RS-IV

During the 4 weeks of the open-label, dose-optimization phase (visit 1 through visit 4), ADHD-RS-IV total scores and subscale scores decreased (improved) from baseline for each LDX dose strength. For the 2 weeks of the crossover period (visit 5 and visit 6), significant reductions in ADHD-RS-IV total and subscale scores from baseline were observed with LDX compared with placebo (by difference in LS means; *P *< .0001). LS mean (SE) change scores for LDX were -25.8 (1.20), -12.5 (0.62), and -13.3 (0.64) for ADHD-RS-IV total, inattention, and hyperactivity/impulsivity scores, respectively. LS mean change (SE) scores for placebo were -8.7 (1.20), -4.1 (0.62), and -4.5 (0.64) for ADHD-RS-IV total, inattention, and hyperactivity/impulsivity scores, respectively.

#### CGI-I scores

At the end of the open-label, dose-optimization phase (visit 4), all subjects (100%) were rated as improved (ie, either very much improved [CGI-I of 1; 64.6%] or much improved [CGI-I of 2; 35.4%]). For the crossover period, 93 (82.3%) subjects were improved on LDX (58.4% very much improved and 23.9% much improved) vs 22 (19.5%) on placebo. Of those subjects, 81 (71.7%) were improved while receiving LDX but not placebo, while 10 subjects (8.8%) were improved on placebo but not on LDX. The overall difference between LDX and placebo treatment was statistically significant (*P *< .0001).

### Safety assessment

No deaths or serious AEs were reported during this study. The most common TEAEs with an incidence ≥ 10% during the dose-optimization phase are reported in Table 4. Most subjects reported mild or moderate TEAEs during this phase; 5 subjects (3.9%) reported severe TEAEs (blunted affect, insomnia, increased insomnia, and irritability each reported in 1 subject; accidental overdose and upper abdominal pain both reported in the same subject). The investigator deemed that the TEAEs were related to study drug in 100 subjects (77.5%).

**Table 4:** TEAEs ≥ 10% During Dose-Optimization and Crossover Phases

**Adverse Event** **Preferred Term**	**Dose-Optimization Phase (Safety Population)**	**Crossover Phase** **(Randomized Population)**	
	**LDX All Doses** **(N = 129)** **n (%)**	**LDX All Doses** **(n = 115)** **n (%)**	**Placebo** **(n = 115)** **n (%)**

Any adverse event	110 (85.3)	38 (33.0)	22 (19.1)

Affect lability	13 (10.1)	0 (0.0)	1 (0.9)

Decreased appetite	61 (47.3)	7 (6.1)	1 (0.9)

Headache	22 (17.1)	6 (5.2)	2 (1.7)

Insomnia	35 (27.1)	5 (4.3)	0 (0.0)

Irritability	21 (16.3)	1 (0.9)	1 (0.9)

Upper abdominal pain	20 (15.5)	2 (1.7)	3 (2.6)

TEAEs were assigned to either the open-label dose-optimization phase or the double-blind crossover phase of the study and were summarized separately. TEAEs that continued uninterrupted from the dose-optimization to the crossover phase without a change in severity were counted only in the dose-optimization phase category. TEAEs with a change in severity across phases or that resolved and then restarted in the crossover phase were counted both in the dose-optimization and crossover arms. TEAEs for which a missing or incomplete start date made it impossible to determine in which phase of the study they started were counted as starting in the dose-optimization phase.

LDX: lisdexamfetamine dimesylate; TEAEs: treatment-emergent adverse events

During the double-blind crossover period, the investigator deemed that the TEAEs were related to study drug in 20 subjects (17.4%) in the LDX group and 8 subjects (7.0%) in the placebo group. There were no new TEAEs with an incidence ≥ 10% during the double-blind crossover period. TEAEs reported during this phase were mild or moderate, with the exception of severe insomnia in 1 LDX-treated subject.

All TEAEs leading to discontinuation occurred prior to participation in the double-blind crossover phase (see Table 1 for a summary of subject disposition). Most TEAEs resulting in discontinuations during the dose-optimization phase were judged to be related to study drug and most were moderate in severity and resolved after discontinuation. TEAEs that led to discontinuations are depicted in Table 5. These TEAEs were judged to be related to treatment and resolved after discontinuation. No TEAEs during the crossover phase resulted in discontinuations. Suicidal ideation, related to study drug after 4 days exposure to LDX during dose optimization and assessed as mild in severity, occurred in 1 subject, an 11-year-old male subject with no other reported clinical conditions at baseline. This subject's suicidal ideation resolved with discontinuation of LDX with no additional reported AEs at follow-up 1 month after discontinuation.

**Table 5:** TEAEs Leading to Discontinuation by System Organ Class and Preferred Term (All Occurrences: Safety Population, N = 129)

**System Organ Class**	**Preferred Term**	**n (%)**
**Gastrointestinal**		4 (3.1)

	Abdominal pain	2 (1.6)

	Diarrhea	1 (0.8)

	Nausea	2 (1.6)

	Vomiting	2 (1.6)

**General**		4 (3.1)

	Fatigue	2 (1.6)

	Irritability	2 (1.6)

**Investigations**		1 (0.8)

	Weight decrease	1 (0.8)

**Metabolic/nutritional**		3 (2.3)

	Anorexia	2 (1.6)

	Decreased appetite	1 (0.8)

**Nervous system**		2 (1.6)

	Amnesia	1 (0.8)

	Psychomotor hyperactivity	2 (1.6)

	Tardive dyskinesia (lip-smacking)	1 (0.8)

**Psychiatric**		5 (3.9)

	Insomnia/sleep disorder	4 (3.1)

	Suicidal ideation	1 (0.8)

	Tearfulness	1 (0.8)

TEAEs: treatment-emergent adverse events

At baseline (n = 129), mean (SD) SBP, DBP, and pulse rate were 100.3 (9.1) mm Hg, 61.2 (7.4) mm Hg, and 84.0 (11.1) bpm, respectively. At the end of the dose-optimization phase (week 4; n = 117), the mean (SD) change in vital signs for all LDX doses combined was -0.3 (10.3) mm Hg SBP, 2.1 (8.0) mm Hg DBP, and -4.3 (12.4) bpm pulse rate. There were no dose-related changes in vital signs in the dose-optimization phase. During the crossover period, vital signs increased slightly from baseline for both the LDX- and placebo-treated subjects. Maximum mean (SD) increases from baseline in blood pressure were 4.2 (9.2) mm Hg for SBP (70 mg LDX group at 8 hours postdose), and 4.7 (8.5) mm Hg for DBP (70 mg LDX group at 8 hours postdose). The maximum mean (SD) increase in pulse was 9.9 (9.8) bpm (70 mg LDX group at 12.5 hours postdose) compared with 6.6 (12.9) bpm for the placebo group and 6.6 (13.6) bpm for all active doses of LDX combined at the same time point. Also, mean (SD) increase in pulse at 8.0 hours postdose was 3.5 (13.7) bpm for the 70 mg LDX group, similar to 4.1 (12.8) bpm for the placebo group and 2.6 (13.0) bpm for all active LDX doses combined at the same time point. Consistent with prior clinical studies of LDX, ECG interval data exhibited no clinically meaningful trends. Heart rate increased slightly from baseline consistent with pulse rate findings. At study baseline, mean (SD) QTcF (Fridericia) interval was 389.0 (17.3) msec in subjects receiving placebo (n = 58) and 392.0 (15.4) msec in subjects receiving LDX (n = 67; all doses). At study end or early termination, mean (SD) QTcF was 389.8 (14.3) msec in subjects receiving placebo (n = 58) and 397.3 (19.5) msec in subjects receiving LDX (n = 66; all doses). At study end, 14 subjects had ≥ 1 abnormal result or abnormal change from baseline in QT, QTcF, or QTcB-Bazett reading after study baseline. However, no subject had a QT, QTcF, or QTcB interval ≥ 480 msec or a change from baseline > 60 msec.

Small decreases in weight from baseline were observed and were consistent with the known effect of psychostimulants [21-23]. Baseline mean weight (SD) in evaluable subjects (n = 128) at visit 1 was 75.1 (18.1) lb. Mean (SD) change from baseline in body weight was -2.5 (6.9) lb (n = 117) at the end of the dose optimization phase and was -2.6 (6.4) lb in evaluable subjects (n = 115) at study end.

## Discussion

This study demonstrated an onset of action for LDX at 1.5 hours (the first postdose time point measured), with duration of efficacy up to 13 hours postdose as assessed during the crossover treatment period. The symptoms of ADHD may extend beyond the school day and continue into afterschool activities and family interactions. This trial is the first to demonstrate duration of efficacy up to 13 hours postdose compared with placebo for an approved oral ADHD stimulant medication and may provide an important treatment option for prolonged ADHD symptom control.

Although pharmacokinetic (PK) data were not collected in this study, data from other studies of adults [24] and children [13] may assist in describing this extended duration of action [25]. In both studies, PK data at the 70 mg dose were reported. In healthy adult volunteers, the plasma concentrations showed intact LDX levels reaching their maximum concentration at 1.1 hours after ingestion and then declined to 0 by 5 hours postdose. However, mean plasma levels of d-amphetamine reached their maximum at 3.7 hours and did not approach 0 until 72 hours following the final dose. In fact, the active d-amphetamine was about 18 times that of the intact LDX when drug absorption was compared [24]. In children, the PK data were collected only up to 12 hours postdose. Since the plasma level of d-amphetamine after administration of LDX 70 mg was 86.7 ng/mL at 12 hours [26], it is reasonable to infer that the d-amphetamine levels would continue to be apparent even beyond the 13-hour time period. Thus, the earlier onset and longer duration of efficacy or therapeutic activity summarized for the present study seem to follow the known PK profile of d-amphetamine and not of intact LDX.

The present study also showed that LDX had a marked and sustained effect on attention, behavior, and math scores, as assessed by the SKAMP and PERMP scales. The therapeutic effects associated with LDX were particularly noticeable with regard to symptoms of inattention, whereby LDX produced sustained efficacy compared with placebo across all time points measured using the SKAMP-A subscale. This study showed significant efficacy and separation from placebo at the first time point of 1.5 hours postdose for the SKAMP-D, SKAMP-A, SKAMP total, and PERMP scales, and at the 2.5-hour time point for SKAMP quality of work subscale. LDX-treated subjects in this study continued to show significant improvement in all SKAMP- and PERMP-measured scales when compared to treatment with placebo at all time points up to and including 13 hours postdose (the last time point assessed).

This study expanded and further refined findings from a previous laboratory school study, due to a larger sample size (129 vs 52), and additional time points assessed predose and 13-hour measurements (-0.5 to 13 hours in this study vs 1 to 12 hours) [13]. In the previous study, treatment effects were observed beginning at 2 hours postdose and continued until 12 hours postdose. In addition, results for CGI-I were similar in these 2 studies, which had similar design and assessment schedules. In the previous study, 74% of LDX-treated subjects were rated as improved (ie, very much or much improved) [13] compared with 82% of subjects in this study who were rated as improved while on LDX during the crossover phase. Consistent with the previous study, LDX was generally well tolerated with most AEs reported as mild to moderate in severity [13]. The AEs reported here were similar in nature to those reported in studies of other stimulants and included decreased appetite, insomnia, headache, irritability, upper abdominal pain, and affect lability [10,13,27-29]. The frequency of newly reported AEs was higher in the noncontrolled phase of this study than is typically observed in stimulant clinical trials for decreased appetite and insomnia. It is unclear why this increased incidence occurred during the open-label phase of this study. It is also unknown if it may be related to the prodrug release profile. However, it should be noted that during the subsequent crossover phase, reported incidence of AEs was generally comparable between LDX and placebo treatments. The rate of discontinuations related to TEAEs was consistent with those seen for other stimulants in similar settings [10,30,31].

Predose time points had high (worsened) SKAMP deportment and attention scores in the early morning hours in both groups. However, higher (worse) ratings were evident in the LDX group compared with the placebo group prior to dosing. While there may be multiple factors that contributed to the higher ratings, the group differences may be, in part, related to residual drug from the previous treatment day as has been seen in another study with an amphetamine-based long-acting stimulant [32]. Recent studies of long-acting stimulants examining multiple daily dose PK parameters suggest that residual plasma drug levels from the previous day's dose are measurable before dosing [24,33].

It is of interest that the onset of LDX efficacy for the SKAMP quality of work scale was an hour later than that seen for the other SKAMP subscales of attention and deportment. This subscale of the SKAMP provides ratings of completeness, correctness, and neatness of assigned work versus other components of attention and interactions with peers and adults during the observation periods. It is possible that the items of this subscale, which are often grouped as a component of SKAMP-A, required a longer interval to separate from placebo.

Modest increases in mean SBP, DBP, and pulse were observed, consistent with the known effects of psychostimulants: typically increases of 2 to 4 mm Hg in blood pressure and 3 to 6 bpm in heart rate are reported [21-23]. As noted earlier, the mean increase in pulse at 12.5 hours postdose for the subjects receiving 70 mg LDX was higher than typically seen. Interestingly, the mean increase in pulse seen at 8.0 hours was lower in this group and in line with pulse changes seen in the placebo group. The present study does not include assessment of PK parameters and, thus, such pharmacologic effects cannot be correlated with relevant blood levels. While 14 subjects had one or more abnormal QT/QTc intervals or abnormal QT/QTc change from baseline, none had an interval ≥ 480 msec, and no clinically meaningful trends were observed in ECG parameters.

Strengths of the study included the use of a controlled laboratory school setting, ratings by trained investigators, and dose optimization, which mimicked dose titration in clinical practice. The blinded design of the crossover phase allowed a more valid assessment of patients at their optimized dosage of LDX versus placebo. Reliability and validity of SKAMP and PERMP scales were other strengths as well as their inclusion as subjective and objective evaluations of efficacy, respectively.

Results should be viewed in light of study design limitations. Consistent with laboratory school study designs, the short treatment duration and assessment phases may provide an underestimate of the number and severity of TEAEs typically seen with lengthier treatment of ADHD. Typical of ADHD studies, subjects with severe comorbid psychiatric conditions were excluded. This may have selected for a study population that under-represents psychiatric comorbidities generally seen in ADHD populations [34]. Where efficacy assessments made during the dose-optimization phase (ADHD-RS-IV and CGI-I) are reported, it should be kept in mind that this was an open-label phase of the study with limitations to interpretation inherent to the unblinded design. While efficacy of LDX compared with placebo was demonstrated up to and including the last time point assessed (13 hours postdose), no measurements beyond 13 hours postdose were captured. Further analysis and future studies will be needed to determine the efficacy of LDX in relieving ADHD symptoms at later time points. Other limitations that may affect the ability to generalize these findings include the uniformly high level of baseline severity of the study subjects, the lack of a group of subjects with inattentive type ADHD, and the under-representation of minority populations.

## Conclusion

In a laboratory school setting, school-aged children with ADHD taking LDX demonstrated significant improvements relative to placebo in ADHD symptoms as measured by the SKAMP (attention and behavior), CGI (improvement of illness), ADHD-RS-IV (hyperactivity/impulsivity and inattention symptoms) (clinician-based), and PERMP (academic productivity). Significant efficacy compared with placebo was observed at each postdose time point, at 1.5 hours, and up to and including 13.0 hours in this study as assessed during the crossover treatment period. This time course is expected based on the PK profile of the known, key therapeutic ingredient, d-amphetamine. These findings suggest that LDX may provide effective symptom control in children with ADHD throughout the day and that continues to afterschool activities and family time.

## Competing interests

SBW is a consultant for Abbott, McNeil, Shire, and the NIMH; has received grant/research support from Addrenex, Eli Lilly, McNeil, Psychogenics, Shire, and the NIMH; is on the speaker or advisory boards for McNeil, the NIMH, Shire, and UCB.

SHK is a consultant, has received grant/research support and honoraria from Shire, Addrenex, Comentis, NIMH, NIDA, NINDS, NIEHS, and EPA.

ACC is a consultant for Shire and Novartis; has received grant/research support from AstraZeneca, Johnson & Johnson, Somerset, Shire, Novartis, Abbott, Eli Lilly, Bristol-Myers Squibb and Neuropharm; has received honoraria from Shire; and is on the speaker or advisory boards for Shire, Novartis, and Bristol-Myers Squibb.

LS is an employee of Shire and is a stock shareholder of Shire, Johnson & Johnson, Pfizer, and Sepracor.

## Authors' contributions

SBW was the principal investigator on this study, made substantial contributions to the conception and design of the study, enrolled patients, participated in data acquisition, analysis, interpretation, and presentation. She was deeply involved in drafting the manuscript and revising the intellectual content. She has given final approval of this version.

SHK was an investigator on this study, enrolled patients, and participated in data acquisition, analysis, interpretation, and presentation. He was deeply involved in drafting the manuscript and revising the intellectual content. He has given final approval of this version.

ACC was an investigator on this study, enrolled patients, and participated in data acquisition, analysis, interpretation, and presentation. She was deeply involved in drafting the manuscript and revising the intellectual content. She has given final approval of this version.

LS was the Senior Director, Global Clinical Medicine for this study and made substantial contributions to the analysis and interpretation of the data. She was deeply involved in drafting the manuscript and revising the intellectual content. She has given final approval of this version.

## Acknowledgements

Statistical support was provided by Jack Schreckengost, PhD, formerly of Shire Development Inc.; editorial assistance was provided by Michael L. Pucci, PhD, of Health Learning Systems, part of CommonHealth^® ^and Barbara Gomez, PhD, formerly of Health Learning Systems, part of CommonHealth^®^; and the study was funded by Shire Development Inc.

The 311 study group comprised: Matthew Brams, MD, Ann Childress, MD, Scott H. Kollins, PhD, Marc A. Lerner, MD, Eliot Moon, MD, John M. Turnbow, MD, Bradley Vince, DO, and Sharon B. Wigal, PhD.
